# Royal Hospital and Home for Incurables, Putney

**Published:** 1919-06-14

**Authors:** 


### ROYAL HOSPITAL AND HOME FOR INCURABLES, PUTNEY

It is Wordsworth who says :—

"He only judges right who weighs, compares
And, in the sternest sentence which his voice
Pronounces, ne'er abandons charity,"

and this was the dominant note of the dainty and excellently arranged luncheon which this most deserving charity held in the hall of the Worshipful Grocers' Company on Thursday, June 5. The function, which, in the absence of the Lord Mayor, who was entertaining the President of the Brazilian Republic, was presided over by Lord Wolverton, lacked none of the delicate adventitious artistry which one psychologist recently so aptly termed "prandial ritual," for the band of the Royal Artillery played during the luncheon, and the interludes between the excellently timed and proportioned speeches were filled with song and humorous music.

The Chairman pointed out that the £8,000 annually derived from the funded capital of the institution looked infinitesimal in comparison with 7,000 millions National Debt. Recent years had been the most difficult since 1854, yet the income had improved on the 1917 figure by some £5,000. Mr. Thomas Wickham, L.C.C., the Chairman of the Board, expressed the satisfaction felt that during the anxious period of the war the inmates of the Home had had good food in plenty assured them. Still, the financial position had been an anxious one, and at one time the bank account showed £9,000 on the wrong side. That incubus had now, however, been shed, and the hope was expressed that, with the era of peace and its attendant prosperity the plans for an extension of the Home's activity could be carried into effect. Wages troubles had also obtruded themselves, needing more than one revision of scales; and the movement among nurses for a shorter working day was sympathetically regarded by the management, who recognised that the working day of the nurse was at present too long. This necessarily meant an increase of staff, and this in turn rendered imperative plans for a home for the nurses. The Board proposed to erect such a home as soon as possible, which should accommodate not only the nurses now at the institution, but the additional number which would result from the growth of the Home's activities. He hoped the public would again come generously to their aid. It had also been decided to apply for a Royal Charter, not only because it would give the institution a better standing in the eyes of the public, but by dispensing with the need for trustees it would make for more economical working. This Charter seemed likely to be granted in a few days. Mr. Charles Cutting, the Secretary, announced that, including the gifts at the luncheon, the contributions totalled £4,896 19s. The amounts include Messrs. Rothschild £210, the Lord Mayor £100, £210 collected by the Ladies' Association, and a number of substantial amounts given anonymously. In a happy and well-conceived speech, Mr. A. Lyle-Samuel, M.P., commended the charity to the warm-hearted public.

